# Asymmetric Introgression and Cryptic Natural Hybridization between Two Species of *Teucrium* Section *Polium* (Lamiaceae) on the Balkan Peninsula

**DOI:** 10.3390/plants13121617

**Published:** 2024-06-11

**Authors:** Dmitar Lakušić, Miloš Zbiljić, Zlatko Šatović, Nevena Kuzmanović, Zlatko Liber

**Affiliations:** 1 Institute of Botany and Botanical Garden, Faculty of Biology, University of Belgrade, Takovska 43, 11000 Belgrade, Serbia; dlakusic@bio.bg.ac.rs (D.L.); nkuzmanovic@bio.bg.ac.rs (N.K.); 2 Department of Botany, Faculty of Pharmacy, University of Belgrade, Vojvode Stepe 450, 11060 Belgrade, Serbia; mzbiljic@pharmacy.bg.ac.rs; 3 Department of Plant Biodiversity, Faculty of Agriculture, University of Zagreb, Svetošimunska 25, 10000 Zagreb, Croatia; zsatovic@agr.hr; 4 Centre of Excellence for Biodiversity and Molecular Plant Breeding (CroP-BioDiv), Svetošimunska 25, 10000 Zagreb, Croatia; 5 Division of Botany, Department of Biology, Faculty of Science, University of Zagreb, Marulićev trg 9A, 10000 Zagreb, Croatia

**Keywords:** asymmetric introgression, cryptic hybrids, geographic patterns, natural hybridization, hybrid swarm, microsatellite, morphometry

## Abstract

In this work, we analyzed the morphology and genetic structure of *Teucrium montanum*, *T. capitatum* and their hybrid *T.* × *rohlenae* from three syntopic populations. A morphometric study showed that the parents and their hybrids exhibited continuous morphological variation, with the hybrid positioned exactly between the parents. Genetic analysis revealed that plants morphologically identified as *T.* × *rohlenae* are fertile hybrids that produce hybrid swarms dominated by later-generation hybrids. This suggests that introgression, rather than speciation, is the more likely outcome of hybridization between these plant species. The extent and direction of gene flow between the two species differed markedly between the three syntopic localities. At the Trilj locality, it was clearly unidirectional, with *T. capitatum* playing the dominant role. At the Sićevo locality, gene flow was slightly asymmetric, favoring the genetic background of *T. capitatum*, while at the Sliven site, it was completely asymmetric in the opposite direction. The extreme case of unidirectional gene flow was observed at the Trilj locality where plants morphologically identified as *T. montanum* could not be genetically distinguished from *T. capitatum*. This suggests that interspecific hybridization occurred long ago, leading to introgression and cryptic hybrids, blurring of species boundaries and generating evolutionary noise.

## 1. Introduction

Hybridization represents one of the fundamental forces of evolution, with the potential to result in a range of outcomes, including hybrid divergence and syntopic speciation, introgression, the formation of hybrid zones, the erosion of species boundaries, and the possible extinction of species through assimilation [1,2]. An assessment of the evolutionary consequences of hybridization can provide valuable insights for predicting species persistence and hybrid speciation, and for the conservation of biodiversity [3]. A central topic in the study of evolutionary processes associated with hybridization is the genetic structure of hybrid swarms. These swarms are defined as complex mixtures of parental forms, the first filial generation offsprings (F_1_ hybrids), backcross types and segregation products [4]. As the barriers to gene flow between parental species depend on the structure of the hybrid swarm [5], determining the genetic structure of hybrid swarms is an important initial step in evaluating the outcomes of hybridization with respect to species emergence and persistence. If the hybrid swarm consists exclusively of sterile or unfit F_1_ hybrids, hybridization has a neutral effect and the species barriers are maintained. Fertile F_1_ hybrids usually give rise to hybrid swarms that are dominated by later-generation hybrids with backcrosses to one or both parents, and F_1_ hybrids are rare or absent. In certain instances, hybrid swarms may be dominated by fertile F_1_ hybrids, which can outcompete later-generation hybrids. This can actually prevent further gene flow between the parental species [3].

*Teucrium* L. is a large genus of Lamiaceae with a remarkable degree of adaptive radiation. The majority of its species are long-lived woody plants (nanophanerophytes and chamaephytes) or herbaceous plants (hemicryptophytes), but some species also belong to the therophyte or geophyte life forms [6]. The genus is known to contain a number of different chemical compounds, including polyphenols (phenolic acids, tannins, flavonoids and phenylethanoid glycosides), diterpenoids, phytosterols, essential oils [7,8,9,10] and fatty acid esters [11,12]. The aerial parts of the plants have been employed in traditional medicine for the treatment of liver and stomach diseases and lung diseases, and as a diuretic. Furthermore, they exhibit antibacterial, anti-inflammatory, antiproliferative and antioxidant effects [8,9,13,14,15]. The genus comprises about 250 species, which are widely distributed across Europe, Asia, America and Australia. The Mediterranean region represents the primary distribution area for the genus, with approximately 96% of all species of the genus occurring there [16,17]. Salmaki et al. [18] suggest that the genus *Teucrium* has a paraphyletic origin and that its diversification commenced during the transition from the middle to the late Miocene.

The genus is currently classified into nine sections based on morphological characteristics [17]. However, these sections do not represent natural evolutionary units as they have not been confirmed in recent molecular phylogenetic studies [18]. Although the general phylogeny of the genus does not fully align with its general classification, some well-separated evolutionary lineages fully correspond to some of the sections defined at the morphological level. This is the case of *Teucrium* Sect. *Polium* (Mill.) Schreber, which includes the morphologically well-defined species *Teucrium montanum* L., *T*. *polium* L. and *T. capitatum* L. [18]. *Teucrium capitatum* is a dwarf shrub that is covered with white-greenish, branched hairs. The leaves are narrowly oblong to narrowly obovate and multiply crenate on both margins, while the bracts are leaf-like to entire. The flowers are arranged in a simple or compound head, with white, rarely yellow, red or purple corollas [19,20]. This species is distributed throughout Europe, from the westernmost point in Spain to the easternmost point in Ukraine. Additionally, it is present in northwest Africa and the Middle East, extending from the Arabian Peninsula in the south to Turkmenistan in the northeast [21]. *Teucrium polium* and *T. capitatum* exhibit considerable morphological similarity. The two species can be distinguished by the height of the habitus, with *T. capitatum* being lower. In addition, *T. capitatum* has a simple inflorescence and less hairy corollas [19]. *Teucrium montanum* is a prostrate dwarf shrub with white, appressed, simple hairs. The leaves are narrowly elliptical, with entire margins, densely hairy below and often glabrous above. The bracts are leaf-like. The calyxes may be hairy or glabrous, and often possess setaceous teeth. The flowers are arranged in terminal heads and have white, cream or yellow corollas [22]. *Teucrium montanum* is a species that is widely distributed in southern and central Europe, extending from the northern Netherlands in the west to Ukraine in the east. Additionally, it is found in Asia Minor and Algeria in North Africa [21]. These three species belong to the same phylogenetic lineage [18], and have been found to hybridize naturally. This phenomenon can be observed in the frequent occurrence of plants with intermediate morphology at sites where *T. montanum* coexists with *T*. *polium* or *T. capitatum*. Hybrids between *T. montanum* and *T*. *polium* or *T. capitatum* in different parts of their distribution are described as hybridogenic taxa: *T*. × *castrense* Verg., *T*. × *bogoutdinovae* Melinkov and *T.* × *rohlenae* K.Malý [23,24].

Although populations at several syntopic localities in the Balkan Peninsula exhibit continuous morphological variation linking the species *T. montanum* and *T. capitatum*, a hybrid between these two species (*T.* × *rohlenae*) has so far been confirmed at only one site through comprehensive morphological [23] and genetic studies [25]. In the genetic study, a newly developed bioinformatics tool called Dig-up Primers [26] was employed to simultaneously identify microsatellite markers (SSRs) in the whole-genome assemblies of *T. montanum* and *T. capitatum*. These newly developed microsatellite markers and the genetic analysis at a single site are highly promising for future studies on the hybridization between *T. montanum* and *T. capitatum*. In addition, the utilization of this novel technique at additional sites within the distribution range of both species will prove to be a valuable tool in elucidating the intricate taxonomy and phylogeography of these closely related species. Therefore, the main aim of this study is to determine, using morpho-anatomical methods and microsatellite markers: (a) Whether intermediate individuals were formed by crossing two species under syntopic conditions; (b) What the reproductive status of the hybridogenic individuals is and whether *T.* × *rohlenae* represents a hybrid species or comprises populations of different generations of hybrids, i.e., hybrid swarms; (c) If the latter is the case, is there evidence of asymmetric hybridization and introgression?; and (d) In which direction and to what extent does introgression occur in different parts of the sympatric zone, i.e., are there different geographical patterns of introgression?

## 2. Results

### 2.1. Morphological Diversification of Syntopic Populations of T. montanum, T. capitatum and T. × rohlenae

**Figure 1:**
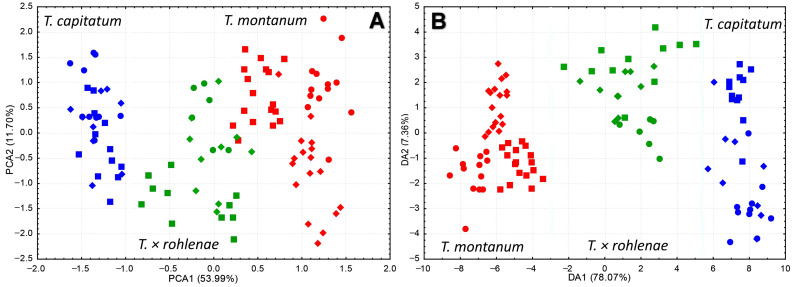
Multivariate analyses for morphometric data for syntopic populations of *T. montanum, T. capitatum* and *T.* × *rohlenae*. (**A**)—principal component analysis (PCA), (**B**)—canonical discriminant analysis (CDA). Each color (red, blue and green) represents a group (*T. montanum*, *T. capitatum* and *T.* × *rohlenae*, respectively), while each symbol (circle, square, rhomb) represents a locality (Trilj, Sićevo and Sliven, respectively).

**Figure 2:**
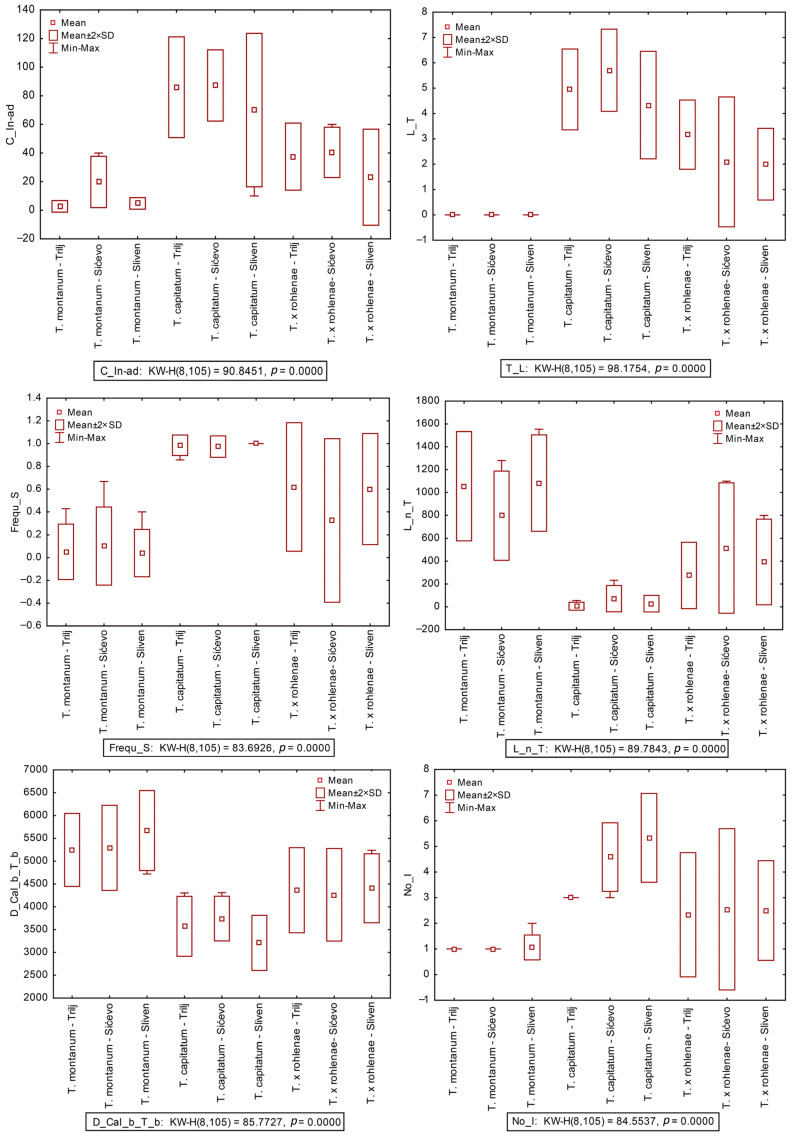
Box-and-whisker plots of morphological characters most contribute to the separation among groups, with Kruskal–Wallis test values. Acronyms: L_T = number of teeth on leaf margin; L_n_T = length of the narrow part of tooth; C_In-ad = indumentum of adaxial leaf surface; Frequ_S = frequency of stipules on stem’s nodes; D_Cal_b_T_b = distance between calyx base and tooth base; No_I = number of terminal inflorescences.

Both principal component analysis (PCA) and canonical discriminant analysis (CDA) were performed on 15 morpho-anatomical data sets for three groups. The results showed that *T. capitatum* and *T. montanum* were completely separated along the first component axis, and that *T.* × *rohlenae* occupied a distinct intermediate position (Figure 1A,B). The results of the discriminant function analysis (DFA) revealed that the following characters were the most influential in distinguishing between the groups: the thickness of the cuticle (T_Cut), the number of teeth on the leaf margin (L_T), the length of the narrow part of the tooth (L_n_T), the indumentum on the adaxial leaf surface (C_In-ad), the leaf surface (L_S), the distance between the calyx base and the tooth base (D_Cal_b_T_b) and the number of terminal inflorescences (No_I) (Table 1, Figure 2).

In all three syntopic localities, the studied groups differed significantly in habitus and in several qualitative and quantitative morphological and anatomical characters (Table S1, Figures S1–S3). *Teucrium montanum* had a cushion-like form with creeping shoots, whereas *T. capitatum* had an erect semi-shrub form with erect lateral shoots. Both habit forms were observed in *T.* × *rohlenae*, which was often determined by the size of the plant. Individuals that were larger and more branched exhibited a true cushion-like form, which is a characteristic of *T. montanum*. On the other hand, smaller specimens usually resembled *T. capitatum* in their habitus. The presence of teeth on the leaf margin was the most striking morphological feature, indicating that the individuals were of hybridogenic origin. These leaf margin teeth were numerous and always present in *T. capitatum*, always absent in *T. montanum*, while there were few of them in *T.* × *rohlenae* or were sometimes even absent. Regarding non-glandular hairs, *T. capitatum* had only multicellular branched hairs, which were completely absent in *T. montanum*. In contrast, the indumentum of *T.* × *rohlenae* consisted of both the branched hairs typical of *T. capitatum* and the unbranched hairs typical of *T. montanum* (Figures S1–S3). The corollas of *T.* × *rohlenae* were similar in size to those of *T. montanum* but had the color of *T. capitatum*.

### 2.2. Genetic Structure and Gene Flow between the Species T. montanum and T. capitatum

A total of 213 alleles were recorded in nine microsatellite loci, of which 155 occurred in Trilj, 141 in Sićevo and 128 in Sliven. A considerable number of alleles (78) were shared between the morphologically determined taxa (Table 2). Private alleles were detected in all three groups and localities (Table 3). The ratio between unique and common alleles in the parental species and hybrids varied considerably depending on the location. For instance, the proportion of private alleles in *T. montanum* varies from 16.41% (Sliven) to 27.74% (Trilj), in *T. capitatum* from 14.58% (Sićevo) to 29.69% (Sliven) and in *T.* × *rohlenae* from 7.81% (Sliven) to 13.19% (Trilj). The largest proportion of common alleles shared by all three groups was recorded in Tilj (14.19%), while the smallest was in Sićevo (8.33%).

The microsatellite diversity parameters showed that all microsatellite loci were highly polymorphic and informative (PIC > 0.700), with values for the information content of polymorphism varying from 0.744 to 0.941 (Table S2).

Further comparison between the studied taxa indicated that the levels of observed heterozygosity *H_O_* and expected heterozygosity *H_E_* were uniform among both the parents and the hybrids. Significant and positive values of the inbreeding coefficient *F_IS_* were only detected in *T. capitatum* from Trilj (0.186), while all other groups showed negative or positive values that were not statistically significant (Table 3).

The proportion of shared alleles distances *D_psa_* showed that the populations from the Trilj were the least genetically differentiated, while those from the Sićevo were the most genetically differentiated. Considering the groups and all localities, the greatest genetic differentiation was observed between *T. capitatum* from Sićevo and *T. montanum* from Sliven, while the most genetically similar groups were *T. capitatum* and *T.* × *rohlenae* from Trilj (Table 4).

**Figure 3:**
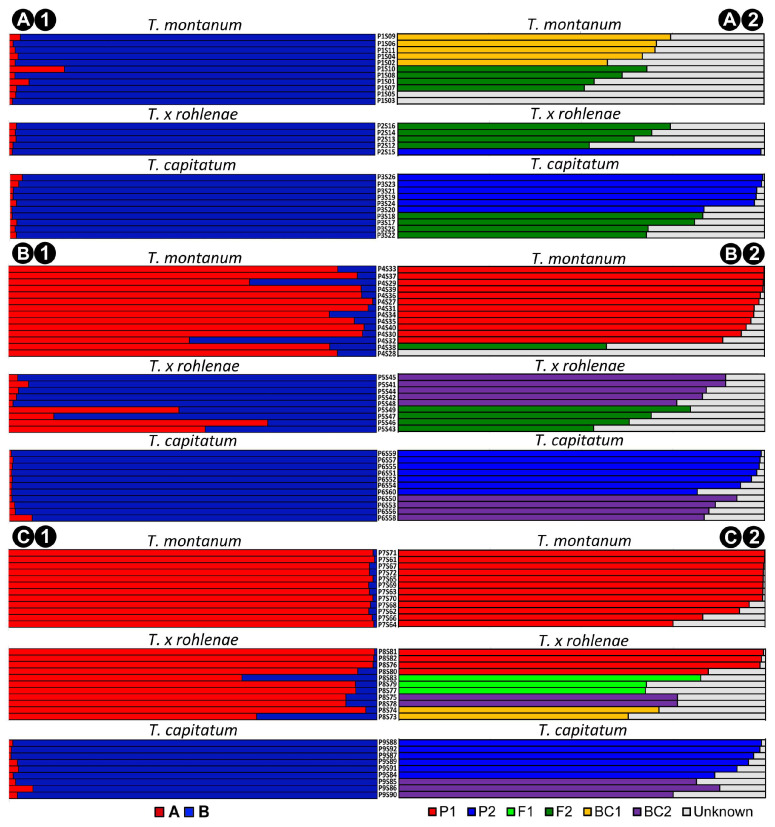
1—The results of the STRUCTURE analysis for the K = 2 based on nine microsatellite loci. All samples were arranged according to morphological identification. Each individual plant is represented by a single horizontal line. The length of the colored segments indicates the estimated posterior probability of assignment of the individual to each class. 2—The genetic structure and classification of individuals from syntopic localities analyzed separately using the program NEWHYBRIDS. Acronyms: P_1_ (parent *T. montanum*), P_2_ (parent *T. capitatum*), F_1_ (first generation hybrids), F_2_ (second generation hybrids), BC_1_ (backcross to *T. montanum*) and BC_2_ (backcross to *T. capitatum*). Each individual plant is represented by a single horizontal line. The length of the colored segments indicates the estimated posterior probability of assignment of the individual to each class. (**A**) For Trilj, (**B**) for Sićevo, (**C**) for Sliven.

As expected, the STRUCTURE analysis yielded K = 2 (ΔK = 24.13) as the most likely number of clusters, with the two genetic clusters corresponding to the parental species *T. montanum* (cluster A) and *T. capitatum* (cluster B). The result of K = 3 (ΔK = 1.11) showed that the hybrids did not form a separate cluster. However, the assignment of individuals morphologically identified as *T. montanum*, *T. capitatum* and *T.* × *rohlenae* was found to be very different at the different localities (Figure 3(A1–C1)).

Regardless of their morphological appearance, all individuals collected at the Trilj locality (Figure 3(A1)) were predominantly assigned to genetic cluster B (*T. capitatum*), with Q values ranging from 0.850 to 0.994.

At the Sićevo locality (Figure 3(B1)), all individuals morphologically identified as belonging to the parental species were assigned to the corresponding genetic clusters. Thus, the average proportion of the membership of *T. montanum* individuals was Q = 0.885 in genetic cluster A, while the average proportion of membership of *T. capitatum* individuals was Q = 0.986 in cluster B. The hybrid *T.* × *rohlenae* individuals were on average assigned to a higher proportion to cluster B (Q = 0.784) than to cluster A (Q = 0.218).

Similar to the case of Sićevo, at Sliven locality (Figure 3(C1)), individuals belonging to the parental species were assigned with a high average proportion of membership to their respective genetic clusters (Q = 0.985 for *T. montanum* individuals in cluster A and Q = 0.979 for *T. capitatum* individuals in cluster B). In contrast to the Sićevo locality, however, the individuals of the hybrid *T.* × *rohlenae* were unambiguously assigned to cluster A, with an average proportion of the membership of Q = 0.901.

The results of the NEWHYBRIDS analyses, which were performed separately for each locality, confirmed the hybridogenic origin of the morphologically intermediate individuals of *T.* × *rohlenae*, in agreement with the results of the STRUCTURE analysis. Individuals assigned to each of the two parental classes (*T. montanum* and *T. capitatum*) and four hybrid classes (F_1_, F_2_ and backcrosses with parental populations) were found in at least one of the three syntopic localities. This indicates that the hybridogenic individuals are fertile and that backcrosses between the F_1_ and F_2_ generation hybrids with the two parents P_1_ and P_2_ have occurred and continue to occur at all three syntopic localities.

As in previous analyses and the NEWHYBRIDS analysis, it was demonstrated that the syntopic populations of certain groups exhibit distinct genetic structures in different locations. Additionally, the direction and intensity of gene flow vary across different parts of the syntopic zone, resulting in diverse geographical patterns (Figure 3(A2–C2)).

At Trilj locality (Figure 3(A2)), of the individuals exhibiting morphological traits indicative of *T. montanum*, none were classified as a parental P_1_ species. Five of the eleven individuals were classified as BC_1_ hybrids, while four as F_2_ generation. Two individuals remained unclassified, with a posterior probability of less than Tq < 0.50 [27] in all categories. Conversely, six out of ten individuals identified as *T. capitatum* were classified as parental P_2_ species, while the remaining four were classified as F_2_ generation. Individuals identified as *T.* × *rohlenae* were classified as F_2_ generation (four individuals) and a single individual was categorized as a parental (P_2_) species.

In contrast to the syntopic population from Trilj, 12 of the 14 individuals of *T. montanum* from Sićevo (Figure 3(B2)) were categorized as parental P_1_ species. One individual was classified as belonging to the F_2_ generation, while the remaining individuals were unclassified. Similarly, seven out of eleven *T. capitatum* were classified as parental P_2_ species, while the remaining four were assigned to the BC_2_ class. No F_1_ hybrids were detected in a group of *T.* × *roheleane* individuals, with five of nine individuals classified as BC_2_ and four as F_2_ generation.

All twelve *T. montanum* individuals at Sliven locality (Figure 3(C2)) were classified as parental P_1_ species, while six of nine *T. capitatum* individuals were classified as parental P_2_ species, and three individuals were classified as belonging to the BC_2_ category. Four of the eleven individuals identified as *T.* × *rohlenae* were also classified as parental P_1_ species, three were assigned to the F_1_ generation, and two belonged to both BC_1_ and BC_2_.

**Figure 4:**
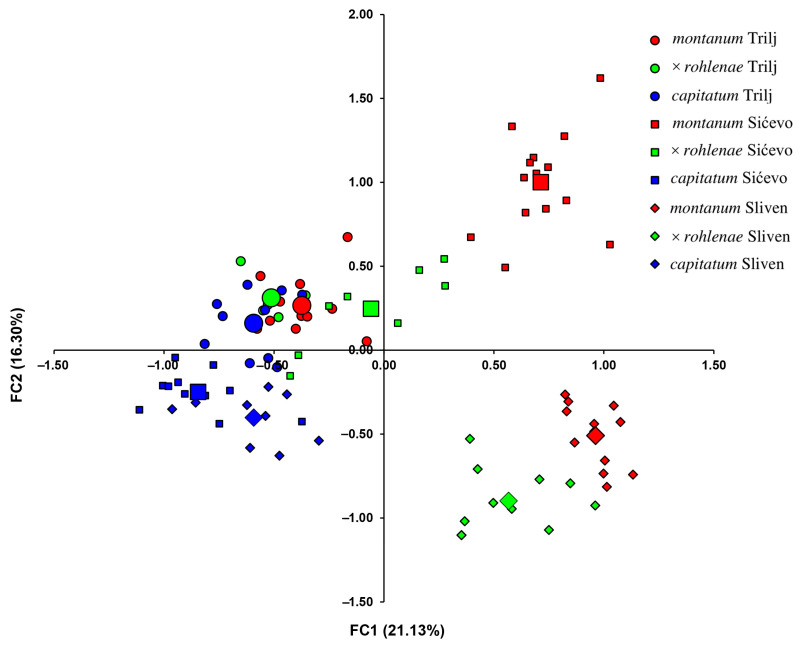
Scatterplot of factorial correspondence analysis (FCA) performed on the basis of genetic data for nine groups. Each individual plant is represented by a small symbol, and enlarged symbols on the scatterplot represent group centroids. Each color (red, blue and green) represents a group (*T. montanum, T. capitatum* and *T.* × *rohlenae*, respectively), while each symbol (circle, square, diamond) represents a locality (Trilj, Sićevo and Sliven, respectively).

The factorial correspondence analysis (FCA) conducted across all three syntopic populations revealed that 37.43% of the total variability is described on the first two axes of the ordination space. The first axis accounted for 21.13% of the total variability, while the second accounted for 16.30%. All *T. capitatum* individuals were positioned in the negative part of the first axis, while *T. montanum* individuals from Sićevo and Sliven were positioned in the positive part of the first axis. At the same time, this analysis demonstrated that, with the exception of the locality Trilj, the majority of individuals exhibiting morphological transition characteristics and identified as *T.* × *rohlenae* occupied an intermediate position between the parental species, thereby corroborating their hybridogenic origin. In addition, the FCA analysis revealed significant geographical differences in the genetic structure of each group at different locations (Figure 4). Thus, all individuals of the parent species *T. montanum* from the three localities were positioned in distinct quadrants of the ordination space. Individuals from Trilj are located in the negative part of the first and the positive part of the second axis (second quadrant). Individuals from Sićevo were positioned in the positive part of the first and second axes (first quadrant), whereas those from Sliven were positioned in the positive part of the first and negative part of the second axis (fourth quadrant). In contrast to the genetically distinct parent species *T. montanum*, the parent species *T. capitatum* exhibited a more uniform genetic structure. All individuals were closely clustered in the negative part of the first axis, with individuals from Sićevo and Sliven overlapping in the negative part of the first and second axis (third quadrant). Individuals from Trilj were located in the spatially proximate part of the first quadrant (negative part of the first and positive part of the second axis), where they completely overlapped with individuals of the parent species *T. montanum* and individuals of *T.* × *rohlenae* from the same locality. The position of the hybrid individuals of *T.* × *rohlenae* varied depending on the classification scheme and the position of the parent species. Consequently, the hybridogenic individuals from Sićevo were positioned in the first three quadrants, occupying a position intermediate between the parent species. The individuals from Sliven were positioned in the fourth quadrant and were quite close to the parent species *T. capitatum*. In contrast, the individuals from Trilj were positioned in the second quadrant and completely overlapped with both parent species (Figure 4).

## 3. Discussion

At all three syntopic localities, plants morphologically identified as *T.* × *rohlenae* were found to be fertile hybrids that produce hybrid swarms dominated by later-generation hybrids, i.e., the F_2_ generation and backcrosses. This suggests that introgression, rather than hybrid speciation, is the more likely outcome of hybridization between these hybridizing taxa. Hybrid swarms consisting only of backcrosses and later-generation hybrids are also common in some other plant groups, such as *Populus* [28] and *Silene* [29].

All analyses performed in this paper indicate that populations from all three localities exhibit distinct patterns of genome flow between parents and hybrid individuals. While hybrids and parents are morphologically distinct in the Trilj locality, they are genetically very similar, with the gene corresponding to the parent *T. capitatum* absolutely dominating all individuals from this locality, including the parent species *T. montanum*. In contrast, the genetic structure in the other two localities differs. Individuals of one parent are dominantly classified as *T. montanum*, and the other as *T. capitatum*, hybrids from Sićevo have a more or less equal share of the genome of both parents, while hybrids from Sliven dominantly possess the genome of the parent *T. montanum*. Based on this, it can be concluded that gene flow in capitatum was found to be unidirectional in one (Trilj) and asymmetrical in the two hybrid swarms (Sićevo, Svilen), as well as that the direction and magnitude of introgression are different in the different parts of the sympatry zone. 

Unidirectional introgression, a phenomenon where back-crosses are realized only with one parental species, has been confirmed in oaks [30], mulberry [31], pines [32] or spruce [33]. Asymmetric introgression, a phenomenon where back-crosses occur with both parental species, but with different magnitudes, has been confirmed in various taxa including rhododendrons [3], pines [32,34], birch [35] or sage [36]. At the same time, different geographic patterns of introgression, which can be recognized in different directions and magnitudes of introgression in different parts of the sympatry zone of hybridizing taxa, were also recorded in some rhododendrons [3] and pines [32,34].

Unidirectional introgression at the syntopic locality Trilj, which is expressed through the dominance of the genome of *T. capitatum* in both parents and the hybrid swarm, is most likely a consequence of the specific dispersion of one of the parental species, which led to the secondary contact of the other parent species and created conditions for unidirectional introgression. Namely, unidirectional introgression occurs when one parent expands its area of distribution to an area already inhabited by another parent that finds its ecological optimum in it. In that case, unidirectional introgression takes place dominantly from the resident species to the invading species [33]. Although both parents (*T. montanum* and *T. capitatum*) have wide ecological valences, the sub-Mediterranean ecological conditions prevailing in the Trilj habitat are much closer to the ecological optimum of the species *T. capitatum* than *T. montanum*, which finds its ecological optimum in cooler, submontane to montane continental conditions [22,37,38]. A possible scenario is that during the glaciation, *T. montanum* may have migrated from montane to lower sub-Mediterranean areas where *T. capitatum* thrives. This may have resulted in a unidirectional introgression, transferring genes from *T. capitatum* to *T. montanum*, leading to genetic homogenization of parents and hybrid swarms. It is also likely that an ancient unidirectional introgression occurred in the wider area of the Croatian coast, as morphologically authentic individuals of *T. montanum* with specific DNA markers of the species *T. capitatum* were also recorded in several populations of *T. montanum* in the area of Istria and Pelješac [22].

Moreover, our findings indicate that cryptic hybridization is present in all three syntopic localities. In particular, a high number of individuals exhibited a “species-specific” morphology of one parental species, yet possessed specific DNA markers of another parental species or later-generations hybrids. This phenomenon was observed with particular frequency in the Trilj locality. At the same time, it is suggested that interspecific hybridization occurred between *T. montanum* and *T. capitatum* long ago, as some plants identified as *T. montanum* had *T. capitatum*-specific DNA markers. Therefore, the genetic structure of syntopic populations is the result of both recent and ancient hybridization events. Cryptic hybridization is a phenomenon where a part of the genome of one parent can pass to the other parent during asymmetric hybridization by hybrid individuals, without significantly affecting the “species-specific” morphology of the other parent. This phenomenon is not uncommon in nature. Backcrossing with one or both parental species can result in individuals that are morphologically difficult to distinguish from the parent species, making the identification of hybrids based on morphology challenging or impossible. This phenomenon has already been observed in numerous plant genera, including *Cardamine* [39], *Betula* [35], *Pinus* [32,34], *Knautia* [40], *Primula* [41], *Ruppia* [42], *Ramonda* [43], etc.

The asymmetric hybridization observed in the Sićevo and Sliven areas may also be related to the habitat conditions to which the syntopic populations are exposed. Namely, it has been established that populations that are outside the ecological optimum in a certain habitat are often considered “sink populations”. This means that genes flow from other species that find more favorable ecological conditions in the zone of sympatry [36]. The ecological conditions at the Sićevo locality are more or less ecologically optimal for both parental species. The locality Sićevo is located within the temperate zone in the hilly-mountainous (at about 500 m above sea level) zone, which represents the optimal zone for the species *T. montanum* [22,37,38]. In addition, the Sićevo gorge is an optimal habitat for the species *T. capitatum*, which due to strong Mediterranean influences, is a suitable habitat for other typical Mediterranean species such as *Salvia officinalis*, *Ruta graveolens*, *Paliurus spina-christi*, etc. [44]. As both parents are more or less in the ecological optimum, there is an almost equal flow of genes between both parents, with a slightly higher proportion of backcrossing between hybrid swarms and *T. capitatum*. At the third syntopic locality (Sliven), the species *T. montanum* reaches its ecological optimum, while *T. capitatum* is in the “combat zone”, i.e., outside its ecological optimum. *Teucrium montanum* has its ecological optimum in thermophilic deciduous forests and on rocky grounds with a continental climate character, i.e., in habitats that are ecologically more similar to the third syntopic locality Sliven. In contrast, *T. capitatum* has its ecological optimum in the Mediterranean rocky grounds (*Cymbopogono-Brachypodietea*) in the Mediterranean and sub-Mediterranean climate zones [37,38]. Therefore, in this locality, there was most likely a gene flow from the parental genome of *T. montanum* in the direction of the hybrid swarms.

The asymmetry of interspecies gene flow is likely caused by a demographic imbalance between the two species at the sympatry zone and differences in optimal or marginal environmental conditions faced by different populations. Nevertheless, in order to gain a more comprehensive understanding of this phenomenon, further research is needed on a larger number of syntopic and allopatric populations. The importance of conducting further more in-depth investigations into hybridization in these species cannot be overstated. While hybridization is an important evolutionary factor that can lead to the emergence of new genotypes, it can also blur the boundaries between species and evolutionary lines [45,46,47]. This was certainly the case with the complex species *Teucrium montanum* s.l. on the Balkan Peninsula.

## 4. Materials and Methods

### 4.1. Plant Material

**Figure 5:**
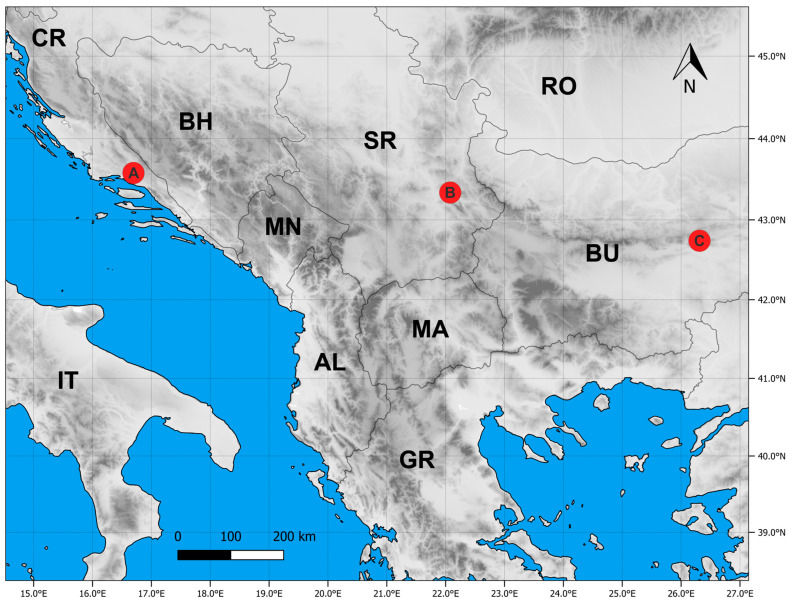
The sympatric zone of the sibling species *Teucrium montanum* and *Teucrium capitatum* in which the presence of the hybrid *T.* × *rohlenae* was registered. Red points: A—Trilj (Croatia), B—Sićevo (Serbia), C—Sliven (Bulgaria). Country abbreviations: CR—Croatia, BH—Bosnia and Herzegovina, SR—Serbia, RO—Romania, BU—Bulgaria, MN—Montenegro, IT—Italy, AL—Albania, MA—North Macedonia, GR—Greece.

All analyses included a total of 92 individuals from three syntopic populations, comprising the parental species *T. montanum* and *T. capitatum*, as well as the hybrid *T.* × *rohlenae*. Samples were collected from their natural habitats in three regions of the Balkan Peninsula, which are ecologically and biogeographically distinct (Table 5, Figure 5). Several specimens were selected for vouchers from each population and were deposited in the Herbarium of the Institute of Botany and Botanical Garden of the Faculty of Biology, University of Belgrade (BEOU) [48]. Between five and fourteen individuals of each parent and hybrid were collected from each locality. The samples for the morphometric study were fixed in the field in a mixture of glycerol and 50% ethanol (1:1). A total of 46 morpho-anatomical characters were measured on each individual. All measurements were performed using Digimizer Image Analysis software 4.6.1 [49]. For the genetic study, young shoots or only leaves (if no young shoots were present) were sampled from each individual, placed in separate paper filter bags to avoid mixing the material, and dried in zip-lock plastic bags with silica gel.

### 4.2. Taxonomy and Nomenclature

It is important to emphasize that in the last century, the species *T. polium* L. and *T. capitatum* L. were considered conspecific, whereas *T. capitatum* was treated as a synonym or subspecies of *T. polium* [19,24]. According to Navarro [17], species *T. polium* and *T. capitatum* are considered separate species, which is also confirmed in Salmaki et al. [18] and relevant checklist [21]. *Teucrium polium* is exclusively limited to the area of SW Europe and NW Africa, while *T. capitatum* (“*T. polium*” sensu auct. balc.) has a wider distribution in the Mediterranean area, western Asia and part of central Asia [21]. It is also important to emphasize that in the investigated syntopic localities where hybrid *T.* × *rohlenae* was recorded, *T. montanum*, as one of the parents, is represented by two morphological groups. The taxonomic status of these groups is unclear. Namely, the populations from Trilj and Sićevo belong to the morphological group “montanum“, while the population from Sliven is included in the morphological group “skorpilii“ [50]. Nevertheless, due to the still unresolved taxonomic relations between the groups designated as “montanum“ and “skorpili“, as well as the more straightforward presentation of the obtained results, in this paper, the names *T. montanum* and *T. capitatum* will be employed for the parents, and *T.* × *rohlenae* for the hybrids.

### 4.3. Morpho-Anatomical Analyses

The anatomical analyses of the leaves were performed on slides prepared according to the standard method for light microscopy [38]. The cross sections of the leaves were made using a Reichert sliding microtome with a thickness of 26 µm and 65 µm. The sections were then cleared in Parazone and subsequently washed thoroughly in water. They were then stained with safranin (1% *w*/*v* in 50% ethanol) and Alcian blue (1% *w*/*v*, aqueous). After staining, all slides were dehydrated and mounted in Canada balsam. A total of 23 anatomical characters were measured, 9 on leaf cross-sections with a thickness of 65 μm and 14 on cross-sections with a thickness of 26 μm.

Prior to scanning at high resolution, leaves, bracts, stems and calyxes were affixed to Graphoscope film with transparent tape. Two leaves, five bracts, one stem and two calyxes were analyzed per individual. The leaves were taken from the central area of the lateral shoots, while the stem selected for morphometric evaluation was taken from one of the lateral shoots creeping on the ground to ensure that the stem length analyzed corresponded to the height of the individuals. A total of 21 characters were measured, including two characters representing the ratio between 2 characters and 20 quantitative characters.

All measured characters are listed with their acronyms in Table S1. The detailed methodology and pattern for the measurement are presented in Zbiljić et al. [50].

### 4.4. Statistical Analyses

Descriptive statistics were calculated for all analyzed characteristics, including the lowest measured value (Min.), the mean value (Mean), the highest measured value (Max.) and the standard deviation (Std.Dev.). As the morphometric characteristics examined did not have a normal distribution, all measured values were log-transformed prior to multivariate analysis. To eliminate a single trait from highly correlated pairs, we conducted pairwise Spearman correlation analyses. We excluded several traits with a Spearman correlation coefficient exceeding 0.85 from the multivariate analysis. We performed a Kruskal–Wallis test for each character to determine the significance of the differences between the groups. Two multivariate approaches were employed to compare all assessed traits and groups, including principal component analysis (PCA) and canonical discriminant analysis (CDA) in Statistica v.8.0 [51]. PCA was used to obtain a general overview of the differences between the groups. CDA was performed on three predefined groups recognized as two parental species (*T. montanum, T. capitatum*) and their hybrid (*T.* × *rohlenae*). To sharpen the distinction between the predefined groups, traits with weak statistical significance (*p*) and H-statistic values of less than 10 were excluded from the CDA analysis. Discriminant function analysis (DFA) was performed to identify the traits that contributed the most to the separation of the groups.

### 4.5. Microsatellite Analysis

Genomic DNA was isolated from 25 mg silica gel-dried leaf tissue using a DNeasy Plant Mini Kit (Qiagen^®^, Hilden, Germany). Microsatellite primer pairs (TmUZ05, TmUZ08, TmUZ09, TmUZ11, TmUZ14, TmUZ20, TmUZ26, TmUZ31, TmUZ32) were used for PCR amplification of nine microsatellite loci [25]. The PCR products were detected via capillary electrophoresis on an ABI3730 DNA analyzer (Applied Biosystems^®^, Foster City, CA, USA). The results of capillary electrophoresis were analyzed using GeneMapper 4.0 (Applied Biosystems^®^). The number of alleles (*N_a_*), polymorphic information content (PIC) and probability of identity (PI) of each microsatellite locus were calculated in Cervus v. 3.0 [52]. Microsatellite data were checked for scoring errors due to stuttering, large allele dropout and the presence of null alleles using Micro-Checker v. 2.2.3 [53]. Null allele frequencies were estimated with the expectation-maximisation algorithm using FreeNA [54].

### 4.6. Intrapopulation Genetic Diversity

Population genetic parameters (the observed heterozygosity, *H_O_*; the expected heterozygosity, *H_E_*; the inbreeding coefficient, *F_IS_*) using microsatellite alleles were calculated using Genepop v. 4.7 [55]. Potential departures from the Hardy–Weinberg equilibrium were investigated with Genepop. The significance level was adjusted after sequential Bonferroni corrections for multiple testing using SAS v. 9.4 [56]. Allelic richness (*N_ar_*) and private allelic richness (*N_par_*) of each population were estimated using HP-Rare v. 1.0 [57].

### 4.7. Population Genetic Differentiation and Structure

Population differentiation based on microsatellite markers was assessed by calculating pairwise FST estimates in FSTAT v. 2.9.3.2 [58]. *p*-values were calculated after 10,000 random permutations. The overall genetic structure was visualized using factorial correspondence analysis (FCA) in GENETIX v. 4.05 [59]. The genetic structure of the populations was assessed using STRUCTURE v2.3.4 [60]. The number of clusters was set from K = 1 to 11 and the analysis was performed thirty times per K. Each run consisted of a burn-in period of 200,000 steps followed by 1,000,000 MCMC replicates using the admixture model with correlated allele frequencies. The calculations were performed on the Isabella computer cluster at the University of Zagreb (Croatia), University Computing Center (SRCE). The optimal number of clusters was determined by calculating ΔK [61] using STRUCTURE HARVESTER v0.6.94 [62]. The results were clustered and merged using CLUMPAK [63].

### 4.8. Hybrid Assignment

We used microsatellite data and NewHybrids 1.1 [27] to assign individual plants from each locality to one of six hybrid classes: two parental classes (*T. montanum* and *T. capitatum*) and four hybrid classes (F_1_, F_2_, and backcrosses with parental populations). Uninformative Jeffrey’s priors were used for both mixing proportions and allele frequencies, without prior information on the hybrid status of individuals. Each run consisted of a burn-in period of 100,000 steps followed by 500,000 MCMC sweeps. Results were based on the average of five independent runs. The assignment of individuals to classes was based on a minimum posterior probability threshold (Tq) of 0.50, as suggested by Anderson & Thompson [27].

## 5. Conclusions

In the sympatry zone of *T. montanum* and *T. capitatum*, plants with intermediate morphology described as hybrid *T.* × *rohlenae* were frequently observed in nature. In cases of coexistence of these two species, the populations exhibited continuous morphological variation linking the species. Furthermore, a formal morphometric study confirmed the intermediate morphological features of the hybrid *T.* × *rohlenae*.

Our findings indicate that plants morphologically identified as *T.* × *rohlenae* are fertile hybrids that produce hybrid swarms dominated by later-generation hybrids. This suggests that introgression, rather than speciation, is the more likely outcome of hybridization between *T. montanum* and *T. capitatum*. Therefore, the concept of “hybrid species” of *T.* × *rohlenae* is not supported. The extent and direction of gene flow between the two species differed markedly between the three syntopic study sites. Thus, the gene flow at the Trilj locality was clearly unidirectional, with *T. capitatum* playing the dominant role. At the Sićevo locality, gene flow can be characterized as slightly asymmetric, with the genetic background of *T. capitatum* being favored over that of *T. montanum*. In contrast, gene flow at the Sliven locality was completely asymmetric in the opposite direction, with *T. montanum* clearly outcompeting *T. capitatum*. The most extreme case of unidirectional gene flow was observed at the Trilj locality, where individuals morphologically identified as *T. montanum* exhibited genetic characteristics indistinguishable from those of *T. capitatum*. This suggests that interspecific hybridization occurred in the distant past, resulting in the introgression and formation of cryptic hybrids, as well as the blurring of species boundaries and generating evolutionary noise.

A demographic imbalance between the two species in the sympatric zone and the fact that different populations face optimal or marginal environmental conditions are the most likely causes of asymmetry in gene flow between the species. However, targeted studies on a larger number of syntopic and allopatric populations are needed to shed more light on this phenomenon.

## Figures and Tables

**Table 1:** The discriminant function analysis (DFA) for 15 morpho-anatomical characters for sympatric populations of *T. montanum*, *T. capitatum* and *T.* × *rohlenae*.

Characters	Acro	Wilks’ Lambda	Partial Lambda	F-remove (8.82)	*P*-Level
Central nerve—radius (µm)	R_CN	0.000127	0.786839	2.77681	0.009071
Indumentum adaxial (percentage of coverage)	C_In-ad	0.000147	0.680441	4.81376	0.000070
Number of capitate hairs	No_CH	0.000135	0.740342	3.59495	0.001267
Thickness of adaxial epidermal cells (µm)	T_Epi-ad	0.000118	0.848545	1.82950	0.083127
Thickness of cuticle (µm)	T_Cut	0.000254	0.392434	15.86904	0.000000
Leaf surface (mm²)	L_S	0.000143	0.696917	4.45764	0.000162
Leaf base width	L_B_L	0.000113	0.883877	1.34664	0.232638
Number of teeth on leaf margin	L_T	0.000219	0.454552	12.29969	0.000000
Bract length	B_L	0.000122	0.817758	2.28428	0.029235
Frequency of stipules on stem’s nodes	Frequ_S	0.000128	0.779433	2.90059	0.006739
Average length of first three internodes	Avg_L_F_I	0.000128	0.776624	2.94815	0.006011
Distance between calyx base and tooth base	D_Cal_b_T_b	0.000141	0.707617	4.23524	0.000274
Length of the narrow part of tooth	L_n_T	0.000216	0.461037	11.98250	0.000000
Number of flowers in terminal inflorescence	No_F_I	0.000128	0.782079	2.85609	0.007499
Number of terminal inflorescences	No_I	0.000127	0.785021	2.80698	0.008437

**Table 2:** The number of alleles (*N_a_*) by groups and localities. OnlyTm—number of unique alleles for *T. montanum*; OnlyTx—number of unique alleles for *T.* × *rohlenae*; OnlyTc—number of unique alleles for *T. capitatum*; TmTx—number of shared alleles between *T. montanum* and *T.* × *rohlenae*; TmTc—number of shared alleles between *T. montanum* and *T. capitatum*; TxTc—number of shared alleles between *T.* × *rohlenae* and *T. capitatum*, All—number of alleles shared by all taxa.

Configuration	Locality			All Localities
	Trilj	Sićevo	Sliven	
	*N_a_*	*N_a_*	*N_a_*	*N_a_*
OnlyTm	43	38	21	29
OnlyTx	16	19	10	14
OnlyTc	27	21	38	22
TmTx	14	21	24	29
TmTc	27	15	5	24
TxTc	6	18	15	17
All	22	12	15	78
Total	155	144	128	213

**Table 3:** Parameters of population diversity. *n*—sample size; *N_a_*—average no. of alleles; *N_ar_*—allelic richness; *N_par_*—no. of private alleles; *N_pr_*—private allelic richness; *H_O_*—observed heterozygosity; *H_E_*—expected heterozygosity; *F_IS_*—inbreeding coefficient; *P*—probability of HW equilibrium (after Bonferroni correction): *** *p* < 0.001; “ns” non-significant values.

Locality	Species	*n*	*N_a_*	*N_ar_*	*N_pr_*	*N_par_*	*H_O_*	*H_E_*	*F_IS_*	*P*
Trilj	*T. montanum*	11	11.778	7.192	8	1.026	0.889	0.910	0.023	ns
	*T.* × *rohlenae*	5	6.444	6.444	4	1.015	0.778	0.867	0.103	ns
	*T. capitatum*	10	9.111	6.067	7	0.908	0.667	0.819	0.186	***
Sićevo	*T. montanum*	14	9.556	5.743	9	0.754	0.746	0.799	0.066	ns
	*T.* × *rohlenae*	9	7.778	5.841	9	0.988	0.877	0.852	−0.029	ns
	*T. capitatum*	11	7.333	5.242	6	0.567	0.768	0.796	0.036	ns
Sliven	*T. montanum*	12	7.222	5.029	2	0.361	0.713	0.766	0.069	ns
	*T.* × *rohlenae*	11	7.111	5.265	1	0.353	0.808	0.818	0.012	ns
	*T. capitatum*	9	8.111	5.918	6	0.640	0.840	0.817	−0.027	ns

**Table 4:** Pairwise *F*_ST_ values (lower diagonal) and their significance (upper diagonal) among populations.

Pop	Locality	Species	P1	P2	P3	P4	P5	P6	P7	P8	P9
P1	Trilj	*T. montanum*		ns	***	***	**	***	***	***	***
P2	Trilj	*T.* × *rohlenae*	0.040		*	**	*	*	**	**	*
P3	Trilj	*T. capitatum*	0.088	0.033		***	***	***	***	**	**
P4	Sićevo	*T. montanum*	0.095	0.139	0.154		***	***	***	***	***
P5	Sićevo	*T.* × *rohlenae*	0.065	0.070	0.087	0.086		***	***	***	**
P6	Sićevo	*T. capitatum*	0.087	0.106	0.117	0.174	0.071		***	**	**
P7	Sliven	*T. montanum*	0.108	0.160	0.179	0.097	0.095	0.193		**	**
P8	Sliven	*T.* × *rohlenae*	0.093	0.096	0.092	0.127	0.078	0.128	0.058		**
P9	Sliven	*T. capitatum*	0.084	0.062	0.050	0.159	0.068	0.077	0.169	0.079	

*p*-values: *** *p* < 0.001; ** *p* < 0.01; * *p* < 0.05; “ns” non-significant values.

**Table 5:** Data on the analyzed populations. Acronyms: State code: BG = Bulgaria, HR = Croatia, RS = Serbia; Alt = altitude (in m a.s.l.) Lat = latitude and Long = longitude (in WGS84).

State Code	Locality	Alt	Lat	Long	Substrate	Habitat
CR	Trilj	340	43.579	16.696	Limestone	Sub-Mediterranean rocky grasslands (*Thero-Brachypodietea*)
SR	Sićevo	429	43.338	22.078	Limestone	Continental rocky grasslands(*Festuco-Brometea*)
BU	Sliven	953	42.74	26.313	Limestone	Mountain rocky grasslands (*Festuco-Seslerietea*)

## Data Availability

The data presented in this study are available on request from all authors.

## Supplementary Materials

The following supporting information can be downloaded at https://www.mdpi.com/article/10.3390/plants13121617/s1, Table S1: Descriptive statistics for all measured characters for the three taxa from the three different localities. Main differential characters are marked in red; Table S2: Population genetics parameters. No—number of alleles; PIC—polymorphic information content; PI—probability of identity; GenBank—GenBank accession; Figure S1: Morphological characteristics of the inflorescence, leaves and anatomical characteristics of the leaves of *T. montanum* (m), *T. capitatum* (p), *T. × rohlenae* (x) in the locality Trilj, Bisko (Croatia); Figure S2: Morphological characteristics of the inflorescence, leaves and anatomical characteristics of the leaves of *T. montanum* (m), *T. capitatum* (p), *T. × rohlenae* (x) in the locality Sićevo (Serbia); Figure S3: Morphological characteristics of the inflorescence, leaves and anatomical characteristics of the leaves of T. montanum (m), *T. capitatum* (p), *T. × rohlenae* (x) in the locality Slivenska planina (Bulgaria).
