# Supplementary material for: Asymmetric Introgression and Cryptic Natural Hybridization between Two Species of Teucrium Section Polium (Lamiaceae) on the Balkan Peninsula
Source: Plants (Basel). 2024 Jun 11;13(12):1617. doi: 10.3390/plants13121617 (PMC11207346; doi:10.3390/plants13121617)
Supplement: Supplementary file 1 [file plants-13-01617-s001.zip › Table S2_Figure S1-S3.pdf]

SUPPLEMENTARY MATERIALS

Table S2. Population genetics parameters. **No** –number of alleles; **PIC** – polymorphic information content; **PI** – probability of identity; **GenBank** – GenBank accession.

Locus	Repeat motif	Size range	No	PIC	PI	GenBank
TmUZ05	$(TC)_{14}$	196-260	27	0.930	0.008	PP001804; PP001805
TmUZ08	$(GA)_{16}$	134-186	24	0.903	0.014	PP001806; PP001807
TmUZ09	$(CT)_{15}$	143-215	29	0.941	0.006	PP001808; PP001809
TmUZ11	$(AG)_{25}$	75-149	29	0.932	0.008	PP001810; PP001811
TmUZ14	$(TG)_{19}$	129-155	13	0.744	0.081	PP001812; PP001813
TmUZ20	$(CT)_{13}$	155-193	17	0.884	0.021	PP001814; PP001815
TmUZ26	$(TC)_{20}$	210-264	26	0.930	0.008	PP001816; PP001817
TmUZ31	$(AT)_{15}$	239-281	21	0.875	0.022	PP001820; PP001821
TmUZ32	$(TC)_{13}$	168-230	27	0.937	0.007	PP001822; PP001823
Average			23.667	0.897		

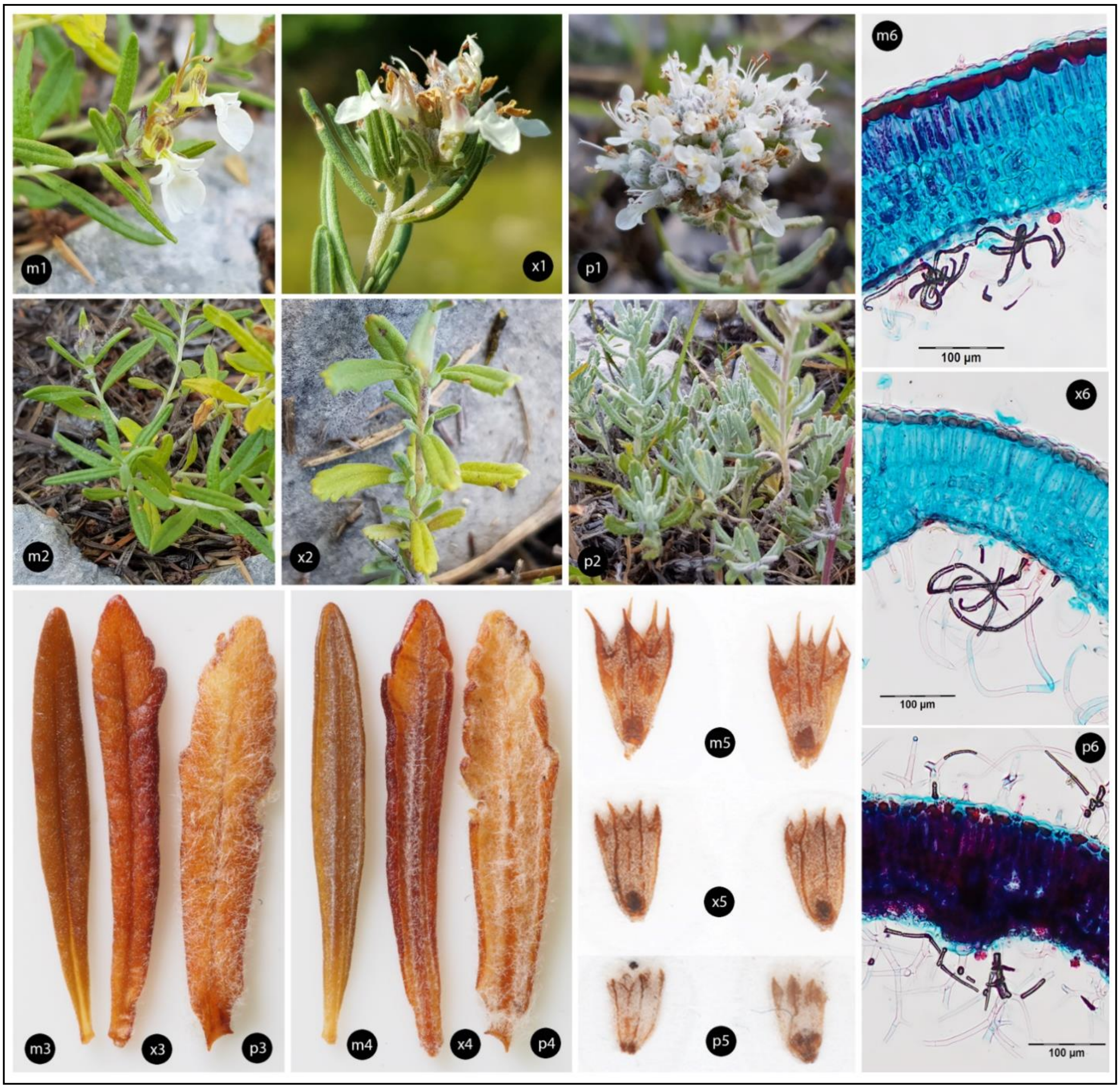

Figure S1. Morphological characteristics of the inflorescence, leaves and anatomical characteristics of the leaves of *T. montanum* (m), *T. capitatum* (p), *T.* × *rohlenae* (x) in the locality Trilj, Bisko (Croatia). 1 – inflorescences, 2 – leaves, 3 – adaxial side of the leaf, 4 – abaxial side of the leaf, 5 – sepals, 6 – transverse section of the leaf.

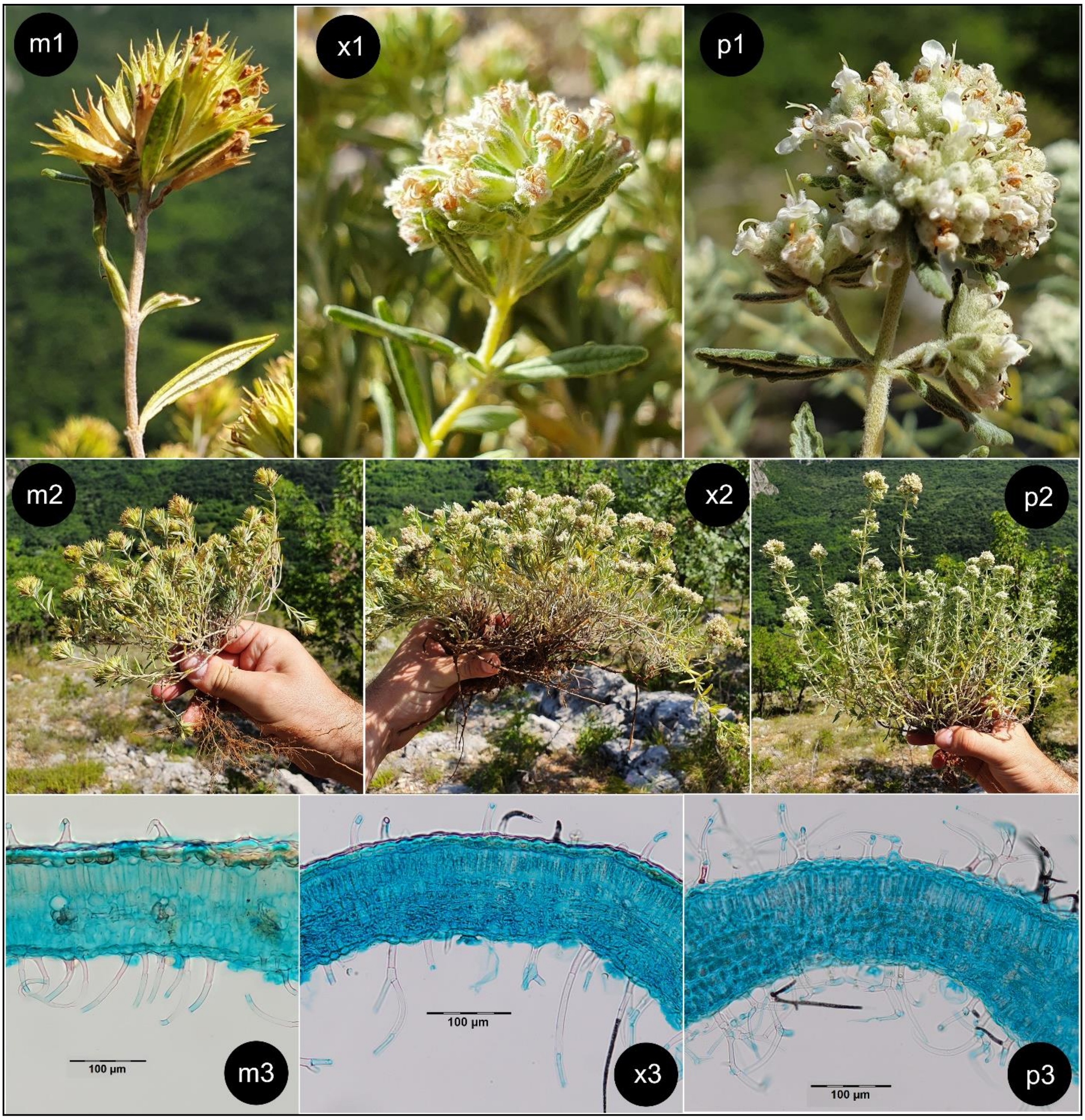

Figure S2. Morphological characteristics of the inflorescence, leaves and anatomical characteristics of the leaves of *T. montanum* (m), *T. capitatum* (p), *T.* × *rohlenae* (x) in the locality Sićevo (Serbia). 1 – inflorescences and leaves, 2 – habitus, 3 – transverse section of the leaves.

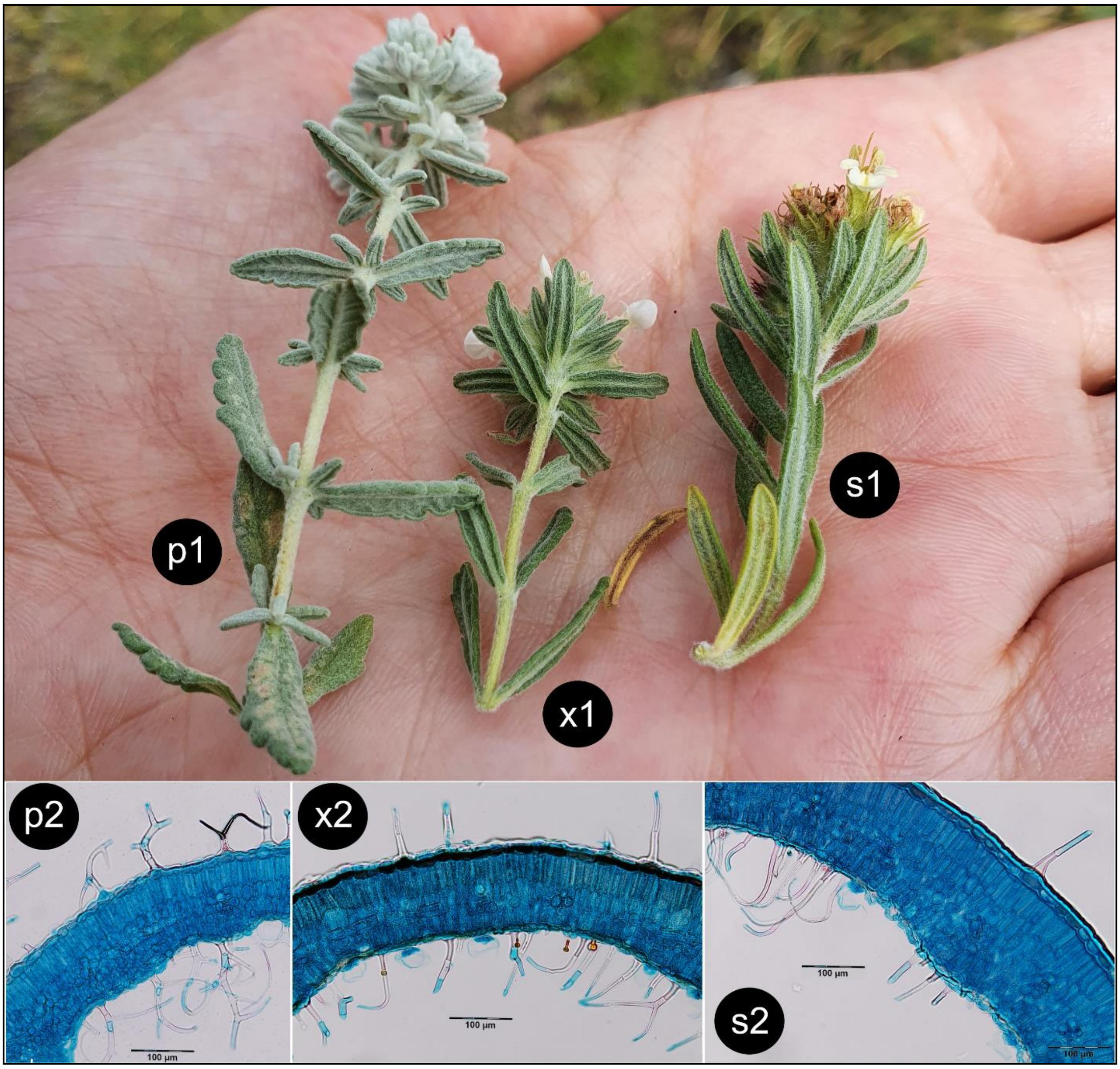

Figure S3. Morphological characteristics of the inflorescence, leaves and anatomical characteristics of the leaves of *T. montanum* (m), *T. capitatum* (p), *T.* × *rohlenae* (x) in the locality Slivenska planina (Bulgaria). 1 – inflorescences and leaves, 2 – transverse section of the leaves.
